# Colon Carcinoma Presenting with a Synchronous Oesophageal Carcinoma and Basal Cell Carcinoma of the Skin

**DOI:** 10.5402/2011/107970

**Published:** 2011-05-03

**Authors:** Nidhi Gupta, Rakesh Kapoor, Suresh C. Sharma

**Affiliations:** Department of Radiotherapy, Postgraduate Institute of Medical Education & Research (PGIMER), Chandigarh 160 012, India

## Abstract

With advances in diagnostic techniques and treatment modalities, the number of patients identified with colorectal carcinoma who develop multiple primary malignancies during long-term followup has been increasing. We report a patient who developed three histologically distinct malignancies. Primary colon carcinoma treated radically followed by an 8-year disease-free period. The patient then presented with progressive dysphagia and was investigated and diagnosed to have a synchronous multicentric squamous cell carcinoma of the oesophagus and basal cell carcinoma of the skin. There was a simultaneous multicentric recurrence in the colon. This case is worth mentioning because the clustering of three primary malignancies (synchronous and metachronous) is of rare occurrence in a single patient, and, to our knowledge, this is the first report of this combination occurring in the same individual. In addition, the report emphasizes the importance of evaluating patients with known colonic primary neoplasms for synchronous colonic and extracolonic tumors.

## 1. Introduction

The phenomenon of multiple primary malignant neoplasms in the same individual was first described by Warren and Gates [1]. Since then, few cases of three or more primary malignant neoplasms have been reported [2]. It is believed that multiple primary malignant neoplasms now occur more frequently than before as a result of prolonged survival of patients after successful treatment of primary tumors. They appear more frequently in the upper digestive tract, respiratory system, head and neck region, or urogenital system. The incidence ranges from 2% to 10% [3]. 

## 2. Case History

A 44-year-old, chronic smoker, nonalcoholic young male underwent exploratory laparotomy, partial colectomy, and end-to-end anastomosis for colon carcinoma, in 1998. Growth was present at the hepatic flexure; there was no metastatic lymphadenopathy, ascites, liver metastases, or peritoneal deposits (T2N0M0). There was no significant family history. Patient did not receive any adjuvant treatment and was lost to followup. 

In August 2007, patient presented with the chief complaints of progressive dysphagia for 3 months, associated with loss of weight and loss of appetite. Patient underwent upper gastrointestinal endoscopy and biopsy which revealed an ulceroproliferative growth at 17-18 cm from the central incisor, and the scope could not be negotiated beyond it. On examination, there was a 3 × 3 cm excoriated lesion over the right cheek with no palpable lymph nodes, and per abdomen examination revealed no palpable mass or organomegaly. 

Endoscopic biopsy from the oesophagus revealed nonkeratinizing-type squamous cell carcinoma. Biopsy from the cheek lesion indicated basal cell carcinoma. Computed tomography (CT scan) of abdomen revealed asymmetric mural thickening in cervicothoracic oesophagus over an approximately 4 cm segment causing near-complete-luminal obliteration. Asymmetrical mural thickening of 1 cm was also seen in the distal 3 cm of oesophagus. There was an annual stricture over a 3 cm segment in the rectosigmoid. There was no significant retroperitoneal or mesenteric lymphadenopathy.

**Figure 1:**
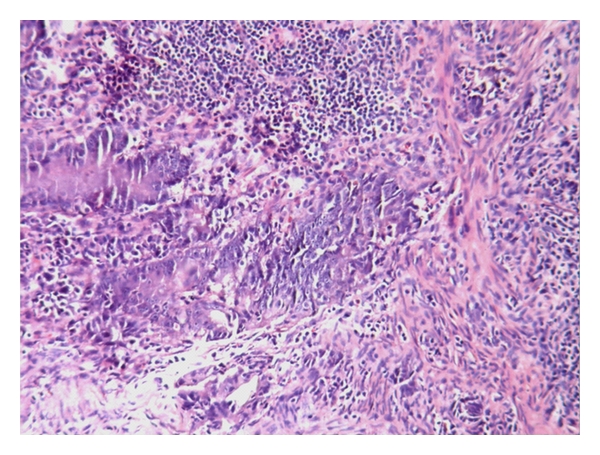
Adenocarcinoma colon.

**Figure 2:**
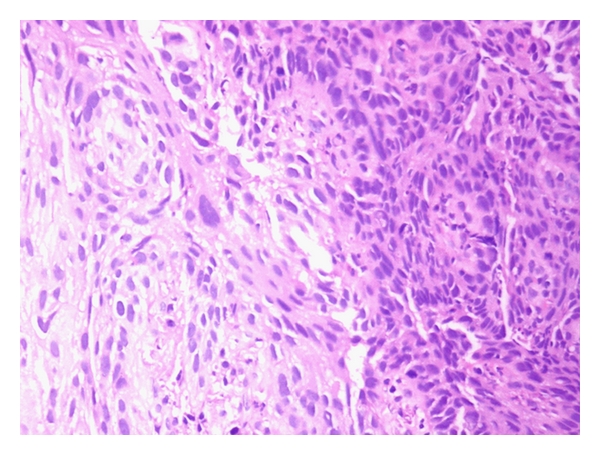
Squamous cell carcinoma of the oesophagus.

**Figure 3:**
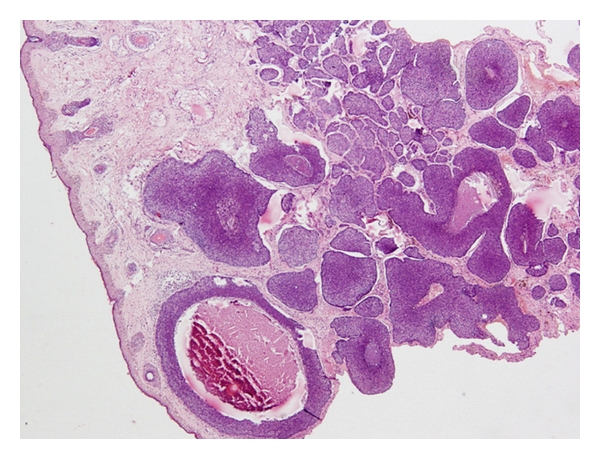
Basal cell carcinoma of the skin.

 Patient underwent colonoscopy and biopsy which showed a fleshy growth at 7 cm from the anal verge, an unhealthy polypoidal growth at 10 cm from the anal verge, and another polypoidal ulcerated growth with stricture at 20 cm. Biopsy from 10 cm and 20 cm growth was suggestive of adenocarcinoma with invasion into muscularis propria. Thus, our patient was simultaneously suffering from multiple malignancies that included adenocarcinoma of the colon (Figure 1), squamous cell carcinoma of the oesophagus (Figure 2), and basal cell carcinoma of the skin (Figure 3). The patient was started on palliative radiotherapy 30 Gy in 10 fractions to upper 1/3 of oesophagus and another 30 Gy in 10 fractions with a bolus to the skin lesion using Cobalt 60 machine. The patient refused colectomy; hence, we started him on systemic chemotherapy for the colon recurrence. After receiving two cycles of chemotherapy with Mayos regimen (5FU and Leucovorin), the patient developed anemia and was asked to arrange donors for blood transfusion but did not turn back. We tried to contact the patient telephonically; the attendants informed that the patient was very weak and was unable to come to the referral institute for any further treatment. Our repeated persuasions failed to convince the family.

## 3. Discussion

Multiple primary malignant neoplasms occur more often in elderly patients, as the incidence of malignancies increases with age. A family history of cancer and genetic predisposition to cancer may be associated with a risk of multiple malignancies [4].

The histological criteria described by Warren and Gates for diagnosing multiple separate primary carcinomas are as follows. (1) Neoplasms must be clearly malignant as determined by histologic evaluation. (2) Each neoplasm must be geographically separate and distinct. (3) The lesions should be separated by normal-appearing mucosa [1].

Synchronous carcinomas are those diagnosed at the same time or within a 6-month period after the diagnosis of the initial cancer. Metachronous carcinomas are secondary cancers that develop 6 months after the diagnosis of the primary cancer, usually after the treatment of the primary lesion [5].

Adenocarcinoma of the colon is the most common visceral cancer in the West, and after the skin and the breast, colon is the most common site for multiple primary malignant tumors. With advances in diagnostic techniques and treatment modalities, the number of patients identified with colorectal carcinoma who develop multiple primary malignancies during long-term followup has been increasing. Extracolonic primary cancer is reported most frequently in skin, stomach, breast, urinary bladder, and prostate. It is shown that the association between different primaries takes place at random and that there are no favourable combinations [6].

Although numerous reports discuss the incidence of synchronous colonic neoplasms, few studies evaluate the relationship between colon cancer and synchronous, extracolonic primary neoplasms. It is estimated that patients with colorectal cancer have extraintestinal primary cancers 1.4 times more often than expected. The incidence of a synchronous, extracolonic primary neoplasm is at least equal to that of a second colonic lesion (between 4% and 5%) [7].

This case is worth mentioning because the clustering of three primary malignancies (synchronous and metachronous) is of rare occurrence in a single patient, and, to our knowledge, this is the first report of this combination (carcinoma of the colon, carcinoma of the esophagus, and basal cell carcinoma of the skin) appearing in the same patient. For our patient, there was no predisposing factor or a family history that might have contributed to the development of these three malignancies except for his being a chronic smoker.

 Through this case report we want to emphasize that it is important for the clinicians to keep in mind the possibility of a metachronous (successive) or a synchronous (simultaneous) malignancy in colorectal carcinoma patients. The possibility of a second or third malignant lesion should be considered in patients with known colon carcinoma. Postoperative long-term screening methods should be established considering the risk of multiple primary malignancies in addition to metachronous colorectal carcinoma.
